# Prognostic significance of senescence-related tumor microenvironment genes in head and neck squamous cell carcinoma

**DOI:** 10.18632/aging.205346

**Published:** 2023-12-21

**Authors:** Young Chan Lee, Yonghyun Nam, Minjeong Kim, Su Il Kim, Jung-Woo Lee, Young-Gyu Eun, Dokyoon Kim

**Affiliations:** 1 Department of Otolaryngology-Head and Neck Surgery, Kyung Hee University School of Medicine, Kyung Hee University Hospital at Gangdong, Seoul, Republic of Korea; 2 Department of Medicine (AgeTech-Service Convergence Major) College of Medicine, Kyung Hee University, Seoul, Republic of Korea; 3 Department of Biostatistics, Epidemiology and Informatics, The Perelman School of Medicine, University of Pennsylvania, Philadelphia, PA 19104, USA; 4 Department of Oral and Maxillofacial Surgery, School of Dentistry, Kyung Hee University, Seoul, Republic of Korea; 5 Department of Otolaryngology-Head and Neck Surgery, Kyung Hee University School of Medicine, Kyung Hee University Medical Center, Seoul, Republic of Korea; 6 Institute for Biomedical Informatics, University of Pennsylvania, Philadelphia, PA 19104, USA

**Keywords:** cellular senescence, head and neck cancer, immunotherapy, microenvironment, single cell

## Abstract

The impact of the senescence related microenvironment on cancer prognosis and therapeutic response remains poorly understood. In this study, we investigated the prognostic significance of senescence related tumor microenvironment genes (PSTGs) and their potential implications for immunotherapy response. Using the Cancer Genome Atlas- head and neck squamous cell carcinoma (HNSC) data, we identified two subtypes based on the expression of PSTGs, acquired from tumor-associated senescence genes, tumor microenvironment (TME)-related genes, and immune-related genes, using consensus clustering. Using the LASSO, we constructed a risk model consisting of senescence related TME core genes (STCGs). The two subtypes exhibited significant differences in prognosis, genetic alterations, methylation patterns, and enriched pathways, and immune infiltration. Our risk model stratified patients into high-risk and low-risk groups and validated in independent cohorts. The high-risk group showed poorer prognosis and immune inactivation, suggesting reduced responsiveness to immunotherapy. Additionally, we observed a significant enrichment of STCGs in stromal cells using single-cell RNA transcriptome data. Our findings highlight the importance of the senescence related TME in HNSC prognosis and response to immunotherapy. This study contributes to a deeper understanding of the complex interplay between senescence and the TME, with potential implications for precision medicine and personalized treatment approaches in HNSC.

## INTRODUCTION

Head and neck cancer (HNC) is the sixth most common cancer, with more than 68,000 patients reported to be newly diagnosed in the United States in 2021 [1]. The 5-year survival rate for HNC ranges from 30% to 70%, contingent upon the tumor's stage and site [2]. This heterogeneity arises from malignant cells harboring diverse genotypes, phenotypes, and interactions within the tumor microenvironment (TME) within each individual tumor, significantly contributing to tumorigenesis and malignant progression, presenting a major obstacle for cancer therapeutics [3]. Cellular senescence, characterized by a state of cell-cycle arrest, can be induced by various profound internal or external stresses, including oncogenic activation or DNA damage from chemotherapeutic agents [4]. The crucial role of senescent cells as a pivotal component of the tumor microenvironment (TME) has been highlighted in Hallmarks of Cancer [3]. Over time, numerous researchers have regarded cellular senescence as a mechanism against malignancy, leading to the conversion of cancer cells into senescent cells [5]. However, emerging evidence in recent years has revealed the dualistic roles of senescent cells, which can either impede or promote tumor development and malignant progression, depending on the specific conditions under which they are induced [6, 7]. In particular, senescent cells secrete several cytokines and growth factors known as the senescence-associated secretory phenotype (SASP). It has been reported that the SASP fosters a close relationship between senescent cells and the TME, inducing immunosuppression and inflammation to promote tumor growth and potentially influencing the response to immunotherapy [8–10]. Moreover, the targeting of senescence process has emerged as a promising strategy in cancer therapeutics [11]. Nevertheless, our current understanding of the interaction between senescence and TME remains limited. Specifically, it remains unclear whether the senescence related TME characteristics observed in patients with head and neck squamous cell carcinoma (HNSC) can serve as predictive biomarkers for clinical prognosis and therapeutic response.

In this study, we identified prognostic senescence-related TME genes (PSTGs) through a comprehensive analysis involving gene-gene network, differential expression analysis, and Cox regression analysis. Furthermore, we conducted clustering analysis using the expression of these genes. Subsequently, we developed a novel risk score model based on senescence related TME core genes (STCGs) to predict patient prognosis and response to immunotherapy. Importantly, the predictive capability of this model was validated in independent cohorts. Additionally, we explored the enrichment patterns of these core genes at the HNSC single-cell level.

## RESULTS

### Identification of prognostic senescence related TME genes

**Figure 1:**
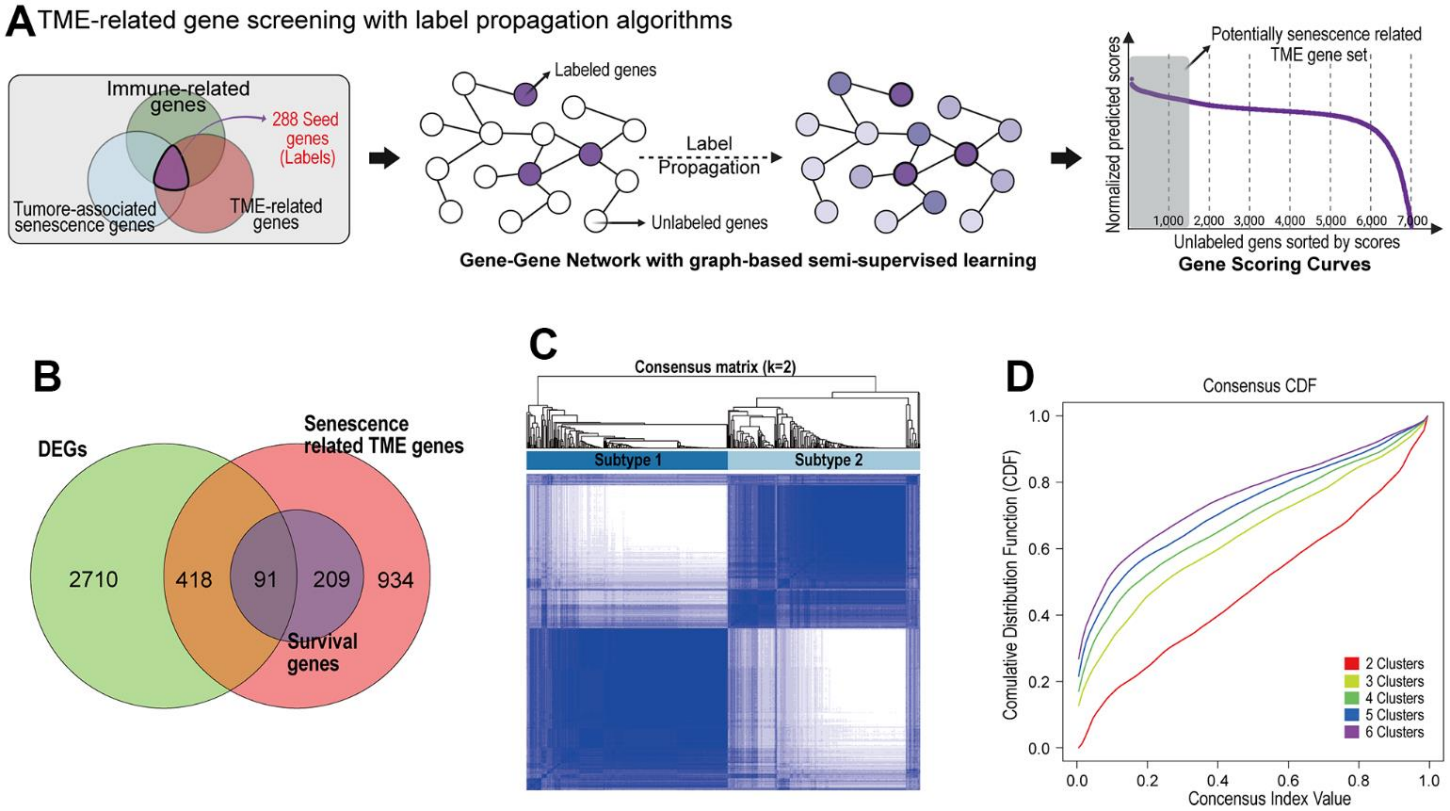
**Identification of prognostic senescence related TME genes and subtype clustering.** (**A**) Senescence related TME gene screening with label propagation algorithms. (**B**) Venn analysis identified overlapping representative gene sets (PSTGs) from differentially expressed genes, senescence related TME gene and survival associated with genes (Survival genes) in Cox analysis. (**C**) Clustering plot of consensus scores for samples in the TCGA–HNSC cohort at k = 2. (**D**) CDF plots corresponding to the consensus matrices in the range k=2,3,4,5,6.

Out of the 7,878 genes, a total of 288 were labeled as nodes when each gene belonged to both categories of TME-related genes, TAS genes, and immune-related genes. The rest of 7,590 genes were set as unlabeled nodes. The predicted gene scores on unlabeled nodes were sorted in descending orders, and the highly scored unlabeled gene set and labeled gene set were selected as the potentially senescence-related TME gene list (N = 1,652) (Figure 1A).

We screened the 3,219 differently expressed genes (DEGs) between HNSC cancer tissue and normal tissues in TCGA-HNSC dataset. To identify the prognostic genes associated with senescence related TME in HNSC, we integrated the DEGs and senescence related TME gene sets, and survival associated with genes (survival genes) in Cox analysis. Finally, we found 91 prognostic senescence-related TME genes (PSTGs). (Figure 1B) [see Supplementary Table 1].

### The identification of senescence related TME subtypes and characterization

Based on the transcriptome expression levels of PSTGs, the Consensus Clustering Method, an unsupervised ensemble clustering algorithm, was employed to cluster the samples in TCGA-HNSC cohort. The consensus matrix CDF curve revealed a distinct flattened segment at K=2, indicating optimal subgrouping (Figure 1C, 1D). Additionally, selecting K=2 for consensus clustering analysis minimized interference between subtypes. A total of 500 HNSC patients in the TCGA database were divided into two subtypes, named subtype 1 and subtype 2.

**Figure 2:**
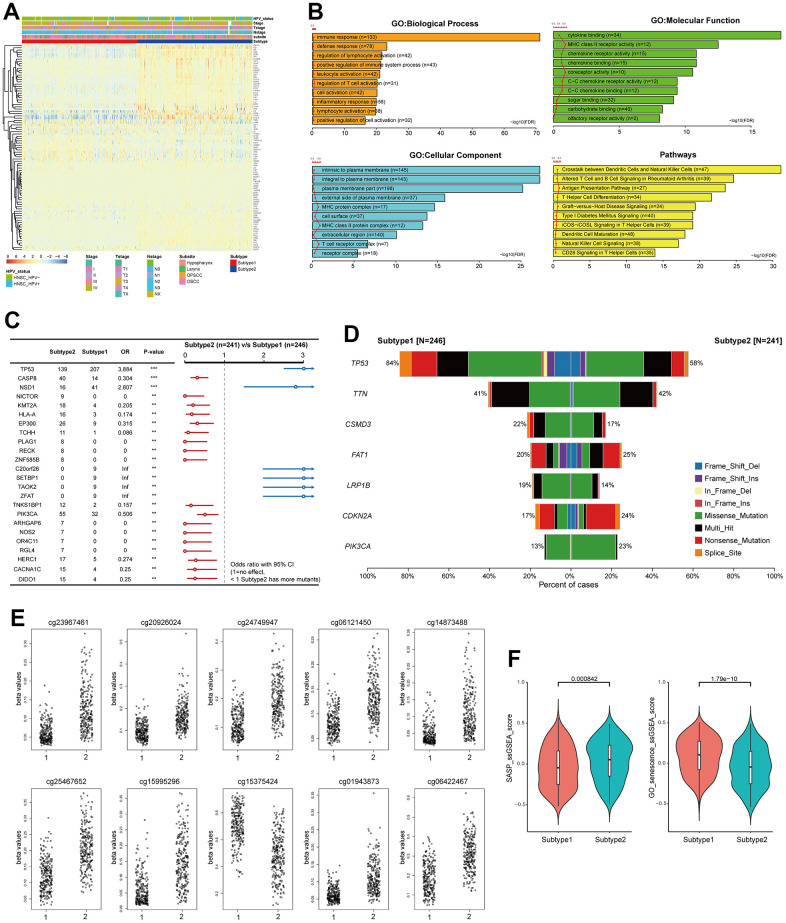
**Distinct characteristics of the senescence related TME subtypes.** (**A**) Heatmap showing the expression of 91 PSTSs of the two subtypes. Red represents high expression and blue represents low expression. Subtype, subsite, stage, T, N and HPV status were used as sample annotations. (**B**) differentially enriched pathway (subtype 2 vs subtype 1). (**C**) Forest plot showing somatic mutation in the two subtypes. (**D**) Barplot showing somatic mutation in the two subtypes. (**E**) Top 10 most significantly differentially methylated CpGs under comparing subtype 1 and 2. (**F**) Differences in SASP and GO_senescence ssGSEA score between the two subtypes in the TCGA–HNSC cohort.

To better understand the characteristics of the two subtypes, the expression levels of PSTGs and various clinicopathologic features were compared between the two subtypes, as illustrated in Figure 2A. The two senescence-related TME subtypes differed in their clinical and molecular characteristics. Subtype 2 demonstrated a higher prevalence of HPV-positive and oropharyngeal cancer cases, whereas subtype 1 was characterized by a higher proportion of advanced T stage, N stage and overall stages.

We conducted an analysis to investigate the presence of differential enrichment in gene ontology (GO) and pathways between the two subtypes based on the DEGs (Figure 2B). The enriched GO terms encompassed biological processes such as immune response and defense response, cellular components including intrinsic and integral to the plasma membrane, and molecular functions such as cytokine binding and MHC class II receptor activity. Additionally, the enriched pathways included crosstalk between dendritic cells and natural killer cells, as well as altered T cell and B cell signaling in rheumatoid arthritis.

We investigated the difference in genomic alteration between the subtypes (Figure 2C, 2D). In subtype 1, the most frequently observed mutation was in *TP53*, found in 84% of the samples, followed by *TTN, CSMD3*, and *FAT1.* On the other hand, subtype 2 exhibited common mutations in *TP53, TTN, FAT1, CDKN2A*, and *PIK3CA*. While *CDKN2A* and *FAT1* mutations were primarily nonsense mutations, the majority of mutations were missense mutations. The TMB was found to be higher in subtype 1 compared to subtype 2 (Subtype1; median 2/MB, Subtype2; median 1.92/MB). *TP53* and *NSD1* mutations were more frequent in subtype 1, whereas CASP8 mutations were more prevalent in subtype 2.

We identified the top 10 most significantly differentially methylated CpGs (p-value ≤0.05; FC >1; Δβ-value >0) when comparing subtype 1 and 2 (Figure 2E). Nine CpG sites (cg23967461, cg20926024, cg24749947, cg06121450, cg14873488, cg25467652, cg15995296, cg01943873, and cg06422467) were hypomethylated in subtype 1 compared to subtype 2, and one CpG site (cg15375424) was hypermethylated in subtype 1. These CpG sites of deregulation of DNA methylation were in *ACVR1, SIL1, TMCO1, MIA2, AGTRAP, CORO1B, HCG20* and *IRF1.* Metascape analysis showed that the genes suppressed by DNA methylation in subtype 1 were enriched in the *ZNF528* target genes among the Transcription Factor Target ontology. SASP and GO_senescence ssGSEA scores were calculated for each sample in TCGA-HNSC to represent the senescence state using the ssGSEA method from the GSVA package, according to the SenMayo gene set and GOBP_MULTICELLULAR_ORGANISM_AGING [12]. It was observed that the SASP ssGSEA score was significantly higher in subtype 2 than in subtype 1 (*p* < 0.05), and the GO_senescence ssGSEA score of the patients in subtype 1 was significantly higher than that of the patients in subtype 1 (*p* < 0.05) (Figure 2F).

**Figure 3:**
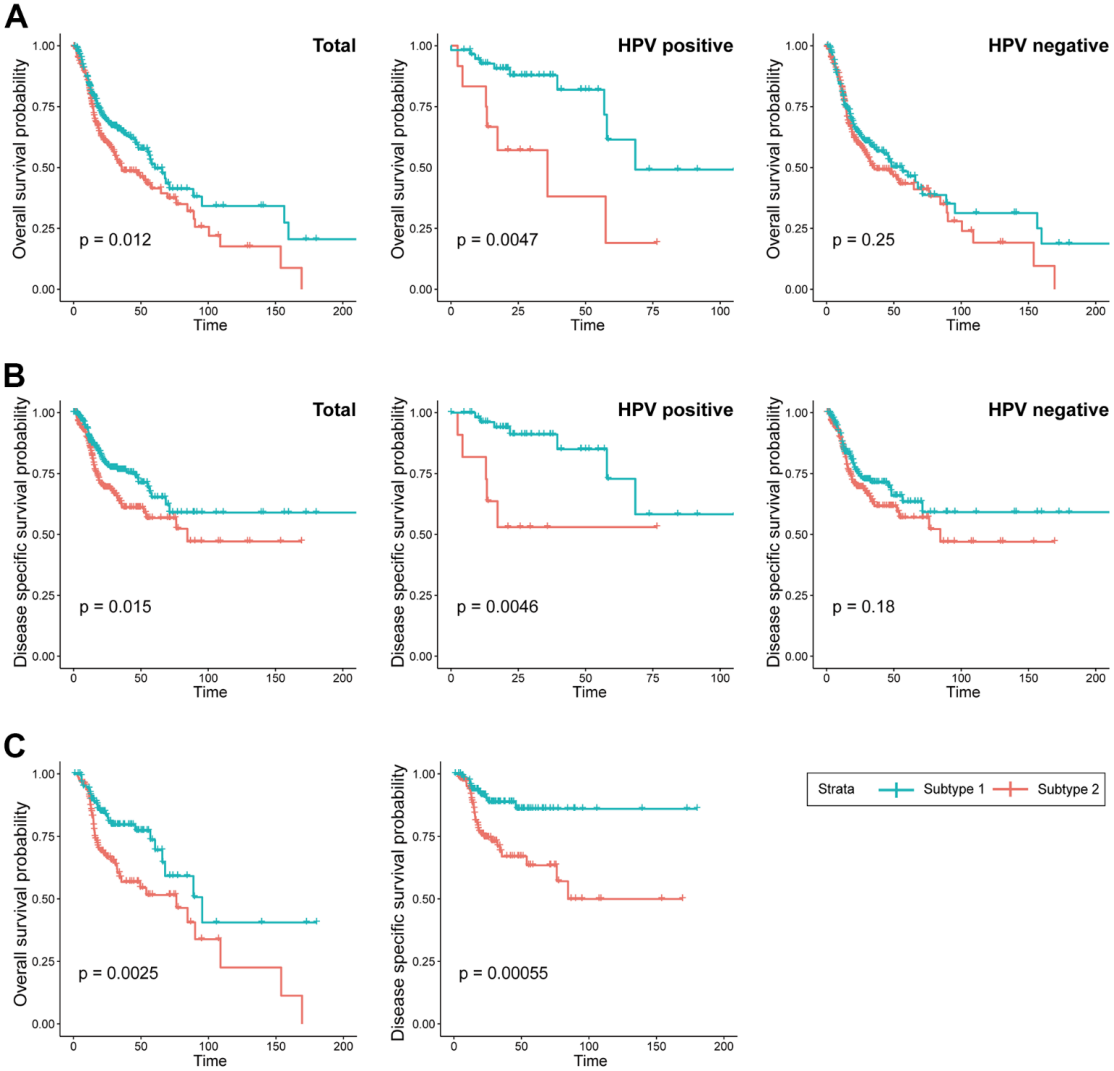
**Prognostic difference between the two subtypes.** (**A**) KM curves indicating prognostic differences in overall survival between the two subtypes in the TCGA-HNSC cohort (Left; total, Middle; HPV positive cohort, Right; HPV negative). (**B**) KM curves indicating prognostic differences in disease specific survival between the two subtypes in the TCGA-HNSC cohort (Left; total, Middle; HPV positive cohort, Right; HPV negative). (**C**) KM curves indicating prognostic differences in overall survival and disease specific survival between the two subtypes in the TCGA-HNSC cohort subgroup who received radiotherapy.

The Kaplan-Meier survival curves revealed significant differences in overall survival (OS) (p = 0.012) between the two subtypes, with subtype 2 demonstrating a more favorable prognosis compared to subtype 1. Furthermore, the significance of the prognostic difference between the clusters was enhanced in the HPV-positive cohort (p = 0.0047), while it diminished in the HPV-negative cohort (p = 0.25). The difference in disease-specific survival (DSS) between the two subtypes exhibited a consistent pattern comparable to that observed for overall survival (Figure 3A, 3B). Remarkably, within the subgroup subjected to radiation treatment, a substantial escalation in prognostic divergence between the two subtypes was observed (OS; p=0.0025, DSS; p=0.00055) (Figure 3C).

**Figure 4:**
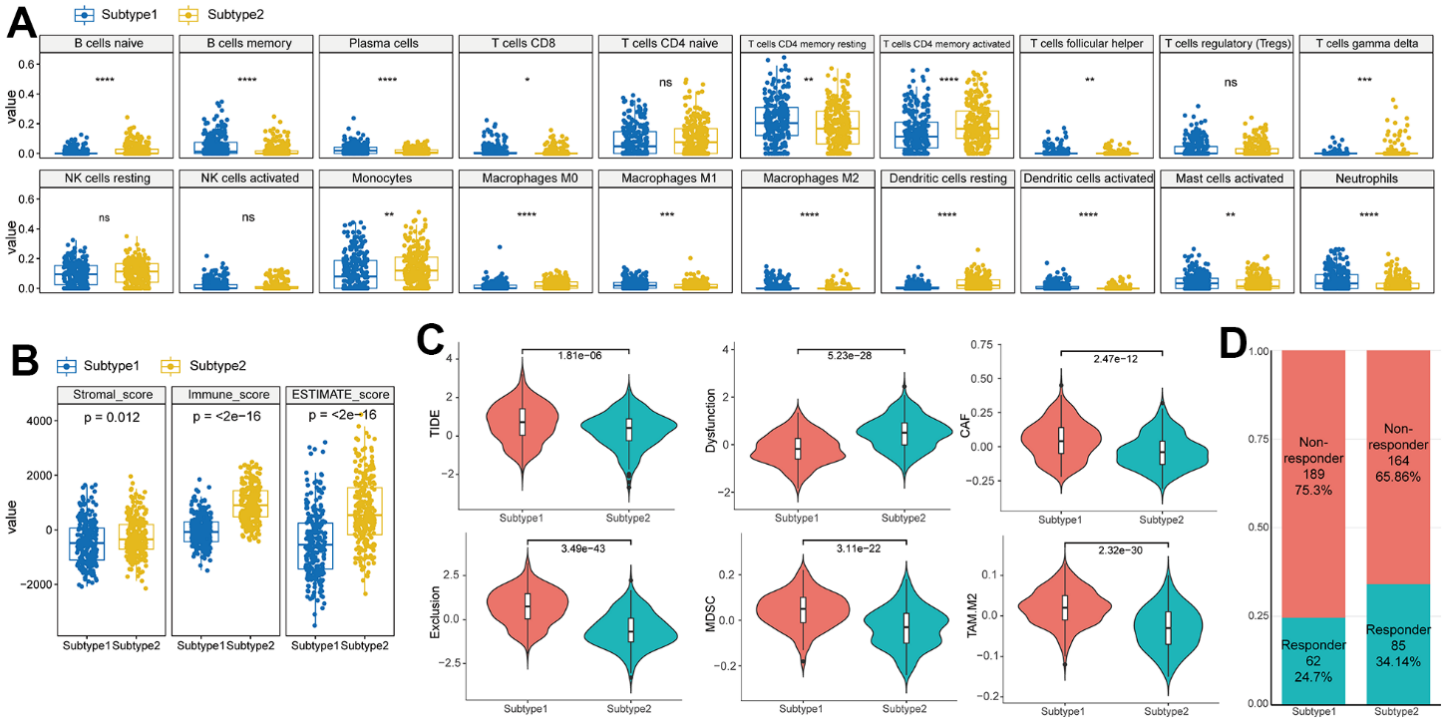
**Difference of immune characteristics between the two subtypes.** (**A**) Differences in immune cell scores between two subtypes in the TCGA–HNSC cohort. (**B**) Differences in ESTIMATE immune infiltration between two subtypes in the TCGA–HNSC cohort. (**C**) Differences in TIDE analysis score between two subtypes in the TCGA–HNSC cohort. (**D**) Differences in immunotherapy response prediction between two subtypes in the TCGA–HNSC cohort.

We utilized immune cell signatures to evaluate the infiltration of immune cells between two subtypes, aiming to assess their distinct immune characteristics. Through the application of CIBERSORT, we discovered significant differences in 16 immune cell types including B cell, CD4 memory activated T cell, macrophages, and dendritic cells between the subtypes (Figure 4A).

To evaluate the characteristics of senescence-related TME subtypes on immunity, we performed the ESTIMATE algorithm. When comparing the immune scores (the proportion of immune cells), stromal score (the proportion of stromal cells) and ESTIMATE score (the proportion of nontumor components) between the two subtypes, we observed that the stromal score, immune score, and ESTIMATE score were significantly higher in subtype 2 (Figure 4B).

TIDE analysis predicted distinct responses to immune checkpoint inhibitors between the two subtypes. Subtype 1 exhibited higher TIDE and Exclusion scores compared to subtype 2, indicating a greater likelihood of immunotherapy evasion. Furthermore, subtype 1 displayed elevated proportions of MDSC, TAM.M2, and CAF, suggesting increased T cell exclusion (Figure 4C). These contrasting immune characteristics between the subtypes led us to hypothesize that they would elicit different responses to immunotherapy. Consistently, the proportion of responders to immunotherapy was significantly higher in subtype 2 (Responder in subtype 1 (24.7%) vs subtype 2(34.1%), p<0.001) (Figure 4D).

### Construction of risk scoring model based on senescence related TME status

**Figure 5:**
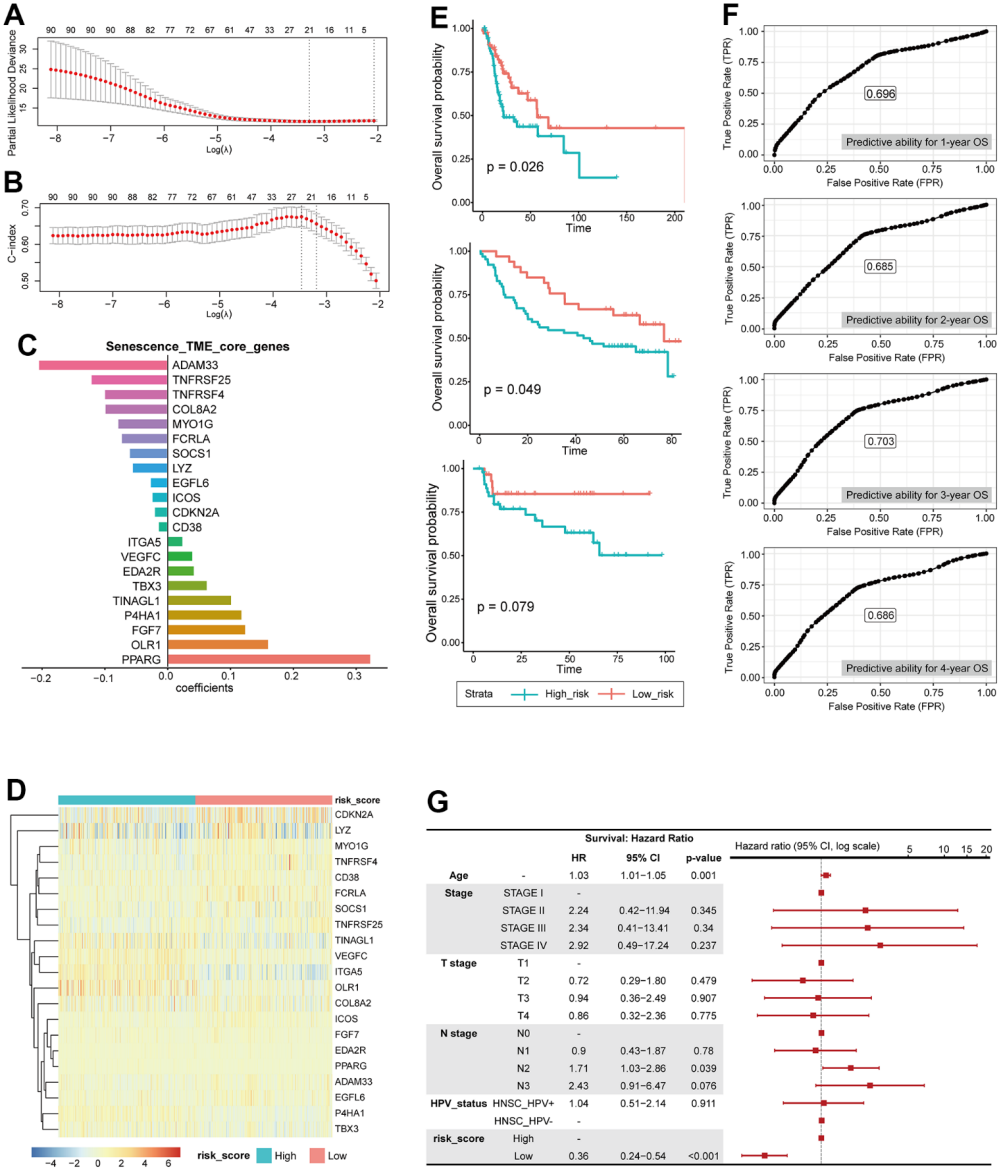
**Construction of risk scoring model based on senescence related TME status.** (**A**) LASSO PLD. (**B**) LASSO c-index. (**C**) core genes, coefficient. (**D**) Heatmap showing the expression of 21 STCGs of the two risk groups. (**E**) KM curves indicating prognostic differences between the two risk groups (Left; HNSC-TCGA test cohort, Middle; GSE41613, Right; KHUMC cohort). (**F**) Receiver operating characteristic (ROC) curves predicting 1-year, 2-year, 3-year, 4-year OS. (**G**) Multivariable analysis with hazard ratio (HR) for OS represented in a Forest plot.

STCGs were selected by performing the LASSO Cox regression algorithm to select penalty coefficient using 91 PSTGs. The minimum log(lambda) was set to the optimal value through 5-fold cross-validation (Figure 5A, 5B).

Finally, several risks (*OLR1, VEGFC, ITGA5, P4HA1, TINAGL1, TBX3, FGF7, PPARG, EDA2R) and protective (CDKN2A, ADAM33, TNFRSF4, SOCS1, TNFRSF25, MYO1G, CD38, FCRLA, EGFL6, ICOS, COL8A2, LYZ*) mRNAs were identified in patients with HNSC (Figure 5C).

The entire TCGA-HNSC set was split into a training set and a test set at a ratio of 7:3. We calculated risk scores based on the expression level of each gene and the coefficient in training set. The median risk score was used as the cutoff value to classify each individual in the TCGA-HNSC cohort as high-risk or low-risk. The heatmap in Figure shows the difference in expression of 21 genes between the two risk groups (Figure 5D).

Prognostic performance of this STCGs based risk model was tested in test set, independent cohorts. Based on the risk model, the test set, GEO cohort (GSE41613), and KHUMC cohort were stratified into high-risk and low-risk groups. Survival analysis demonstrated that patients in the high-risk group exhibited significantly lower overall survival compared to those in the low-risk group in the test set (high-risk (n=49) vs low-risk (n=48), log-rank test, p-value=0.026) and the GEO cohort (high-risk (n=33) vs low-risk (n=64), log-rank test, p-value=0.049). Furthermore, the low-risk group demonstrated a trend towards higher overall survival in the KHUMC cohort (independent cohort, Asian ancestry) (high-risk (n=29) vs low-risk (n=46), log-rank test, p-value=0.079), although it did not reach statistical significance (Figure 5E).

We also calculated the predictive ability for 1-year, 2-year, 3-year, 4-year OS using ROC analysis, and the area under the ROC curve was 0.696, 0.685, 0.703, and 0.686, respectively (Figure 5F). In multivariate Cox regression analysis, the risk score was the most important independent prognostic factor to predict OS (HR: 0.36, CI: 0.24- 0.54, p <0.001) (Figure 5G). Therefore, it was confirmed that senescence related TME risk score can be used as a prognostic biomarker for HNSC.

### Immunotherapy response prediction of senescence related TME risk model and STCGs expression in single cell level

**Figure 6:**
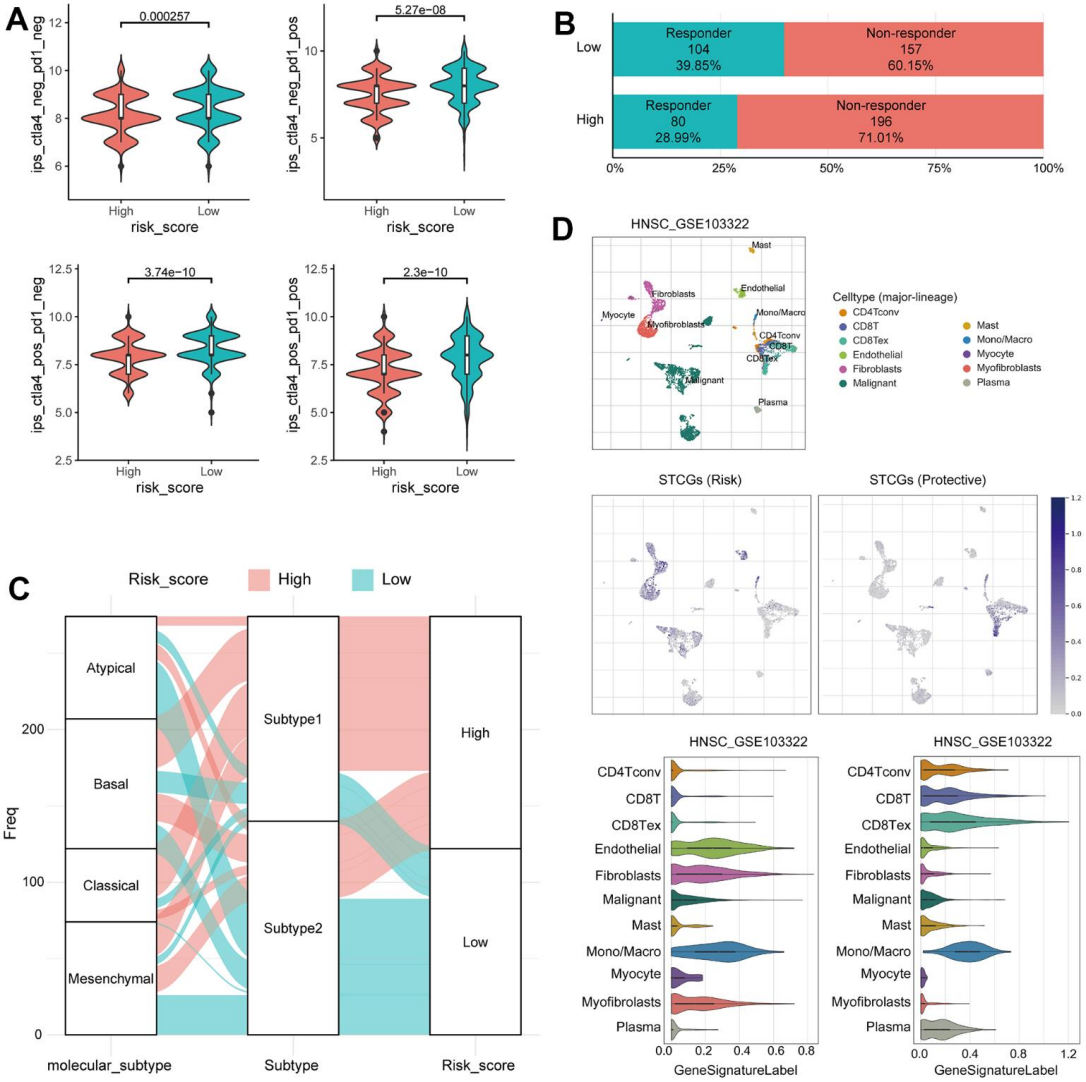
**Immunotherapy response prediction of senescence related TME risk model and STCGs expression in single cell level.** (**A**) Differences in immunophenoscore between two risk group in the TCGA–HNSC cohort. (**B**) Differences in immunotherapy response prediction between two risk group in the TCGA–HNSC cohort. (**C**) Alluvial plot showing the changes of molecular subtype, senescence related TME subtype and risk group. (**D**) Single cell profiling of senescence related TME core genes.

In previous studies, the IPS has demonstrated its predictive value for immune checkpoint inhibitor (ICI) response in melanoma patients, owing to its high immunogenic potential [13]. In our investigation, we observed distinct IPS patterns between the two risk groups among HNSC patients. Specifically, the low-risk group exhibited significantly increased IPS scores in CTLA4 negative/PD-1 negative, CTLA4 negative/PD-1 positive, CTLA4 positive/PD-1 negative, and CTLA4 positive/PD-1 positive. Furthermore, when utilizing the TIDE score to predict ICI response, we observed a significantly higher proportion of responders in the low-risk group (Figure 6A, 6B). These findings suggest that HNSC patients with a low risk of senescence EMT represent promising candidates for ICI therapy. Alluvial diagram showed the mutuality of the molecular subtypes [14], subtype and risk score group in TCGA-HNSC cohort. Mainly, basal and classical subtypes were associated with Subtype 1 and high-risk score group. The atypical subtype associated with HPV positive has a significant correlation with subtype 2 and low risk score group (Figure 6C).

The TME, consisting of immune cells, inflammatory cells, and stromal cells, exerts a crucial role in the initiation, progression, metastasis, recurrence, and acquisition of drug resistance in tumor. Therefore, to further evaluate the senescence related TME characteristics at the single cell level, we investigated the single-cell transcriptome of GEO dataset from primary HNSC tissue. In UMAP and violin plots, STCGs were enriched in fibroblast, mono/macrophage, and T cells rather than cancer cells, suggesting that these cell types, and not cancer cells, contribute to the senescent features of HNSC. Interestingly, expression of risk genes was increased in fibroblast and endothelial cells, and protective genes were mainly expressed in T cells (Figure 6D). In short, the above results suggest a significant association between STCGs and HNSC, with a particularly notable relationship observed in stromal and immune cell populations.

## DISCUSSION

The long-lasting impact of senescent cells on tissue homeostasis has gained prominence, particularly with the identification of the SASP [15]. SASP serves a dual role by not only reinforcing cellular senescence through autocrine signaling but also mediating paracrine effects [16]. Through paracrine signaling, SASP factors have the capability to remodel tissues, impacting the proliferation and migration abilities of neighboring cells such as stromal cells, immune cells, and cancer cells [4, 17]. In addition, SASP factors possess the potential to stimulate angiogenesis and augment the immunosuppressive microenvironment [5]. Coppé et al. found that SASP factors selectively act on immune cells and stromal cells present in the TME, triggering paracrine senescence [18]. This process promotes cancer cell epithelial-mesenchymal transition (EMT) and enhances invasiveness. However, the knowledge regarding the link between cellular senescence in the stroma and TME has remained significantly limited thus far. Furthermore, the impact of cellular senescence associated TME on the efficacy of cancer treatments, including immunotherapy, and its potential as a prognostic indicator remains elusive. To the best of our knowledge, this is the first study to offer a comprehensive evaluation of the senescence related TME status by integrating senescence related TME genes through a gene-gene network and clustering. Furthermore, we have introduced a novel risk model that utilizes a selected gene set to predict prognosis and confirmed the expression of STCGs in immune cells at single-cell levels.

Initially, we identified a set of genes from the intersection in the lists of TAS genes, TME related genes, and immune-related genes. Leveraging these genes as seed nodes, we successfully derived interconnected gene lists within the same network through gene-gene interactions. This proposed model holds the potential to enhance the accuracy and efficacy of selected genes associated with senescence in the TME.

Through the application of consensus clustering using PSTGs, we identified two distinct subtypes. These subtypes exhibited significant differences in mutation profiles, methylation patterns, immune profiles reflecting the TME status, prognosis, and immunotherapy response. Subgroup 1 is more aggressive compared with tumor in subgroup 2. Interestingly, in the analysis of HNSC patients who received radiotherapy, the prognosis of patients in subgroup 1 was worse, and the difference in prognosis between subtypes increased more significantly. This observation suggests that the expression of radiation-induced senescence related genes may have implications for prognosis in the context of this study. The higher mutation rate of the TP53 gene may be implicated in subtype 1 concerning senescence related TME. The loss of p53 function promotes chromosomal instability, leading cells to undergo either senescence or apoptosis through direct and indirect mechanisms [19]. Numerous studies have indicated the significance of DNA methylation patterns in senescence as a pivotal factor influencing tumor behavior [20, 21]. In our results, subtype 1 patients had significantly hypomethylation in several CpG sites than subtype 2 patients and methylation-silenced genes in subtype 1 were enriched in transcription factor target, especially ZNF528 target genes. The data suggest that epigenetic silencing of ZNF528 could be an important factor in the determination of senescence related TME subtype in HNSC.

Furthermore, we observed the significant difference of immune cell profile and TME status between the two subtypes. The results showed that subtype 2 showed significant increases in the infiltration of immune cells such as the activated CD4+ T cells, B cell and macrophages. In addition, there was a significant difference in stromal score, immune score and ESTIAMTE score between two subgroups. The results of this study provide evidence supporting the association between senescence related TME subtypes and distinct TME features. In this context, analysis of functional differences in DEGs indicates that subtype 2 is closely related primarily to immune responses, defense responses, regulation of lymphocyte activation, and crosstalk between dendritic cells and natural killer cells. Interestingly, in the comparison of ssGSEA between the two subgroups, the SASP and senescence gene sets exhibited contradictory findings. It is well-established that senescent cells typically release SASP factors, however, we observed a decrease in SASP expression in subtype 1, characterized by high expression of senescence gene sets. This discrepancy can be attributed to the fact that SASP-associated genes may predominantly reflect immune cell activity within the tumor microenvironment (TME). Conversely, the upregulation of the senescence gene set likely arises from an augmented occurrence of cellular senescence or impaired immune-mediated clearance of senescent cells [22].

Furthermore, we established STCGs including twenty-one genes (*OLR1, VEGFC, ITGA5, P4HA1, TINAGL1, TBX3, FGF7, PPARG, EDA2R, CDKN2A, ADAM33, TNFRSF4, SOCS1, TNFRSF25, MYO1G, CD38, FCRLA, EGFL6, ICOS, COL8A2, LYZ*) were selected by performing the LASSO Cox regression algorithm for predicting the prognosis and therapy response of HNSC patients. Previous studies have provided some level of understanding regarding the biological functions of the genes encompassed in STCGs. Peroxisome proliferator-activated receptors (PPARs) are ligand-activated transcription factors that belong to the nuclear hormone receptor superfamily [23]. *PPARG* is a key regulator of lipid metabolism in diverse immune cells, thus exerting a significant influence on immune regulation [24, 25]. Notably, recent investigations have revealed an unfavorable prognostic association between overexpression of *PPARG* and certain cancer types (e.g., prostate cancer, esophageal cancer), particularly those linked to obesity [26, 27]. Given the highest coefficient of PPARG as a risk core gene in our findings, further exploration into the interplay between lipid metabolism and the senescence process in cancer is warranted [28]. Furthermore, consistent with our findings, previous studies have reported that *OLR1* and *FGF7* are associated with adverse cancer prognosis due to alterations in immune response and TME status [29–31].

In this study, we have validated the predictive capability of the risk score obtained from STCGs across two independent cohorts. This validation emphasizes the robustness and reliability of the model based on STCGs. Notably, a significant disparity in HPV status was observed between the two classified subtypes, leading to the anticipation that our risk model would solely exhibit efficacy in HPV-positive cancer. Surprisingly, in the validation dataset (GSE41613), our developed model demonstrated prognostic capability even in HPV-negative oral cavity cancer patients. Furthermore, in the dataset comprising Asian patients (KHUMC cohort) with different ancestral backgrounds than the TCGA cohort, it was observed that the risk score differentiated the prognosis, although statistical significance was not attained.

To evaluate the expression of STCGs in TME cells, we identified TME cell populations using scRNA-seq data. We found a particularly high expression of risk STCGs in fibroblast and endothelial cells. The bioactive Vascular endothelial growth factor (VEGF) secreted by senescent fibroblasts has a significant impact on tumor angiogenesis and the progression of cancer [32]. Additionally, studies have demonstrated that senescent endothelial cells contribute to the enhanced aggressiveness of breast cancer cells [33]. Thus, our findings strongly suggest that cellular senescence in these specific cell types within TME exhibits pro-tumor properties. Conversely, a notable upregulation in the expression of protective STCGs was observed specifically in T cells, indicating the potential of these immune cells to exert tumor control mechanisms via senescence.

Our study has several limitations. Firstly, despite utilizing public data for the development and validation of risk models associated with senescence, it is imperative to validate these models using prospective data from multicenter studies to enhance their applicability. Secondly, the transcriptional profiles analyzed in this study were generated on RNA sequencing, and the gene expression of the identified STCGs were further confirmed in single cell RNA sequencing in HNSCC tissues. However, several protein products in the risk model were not validated in either HNSCC cell lines or HNSC tumor tissues. Our future research will contain some experiments such as immunohistochemical testing or western blot for expression of STCG in HNSCC tissue. Thirdly, while a risk model based on STCGs has been established, there is a need to improve its diagnostic performance by integrating relevant clinical parameters. Finally, although hypotheses regarding the functions and mechanisms of senescence-associated genes within the TME have been proposed, further comprehensive investigations are essential to unravel the specific mechanisms underlying their actions.

In conclusion, this study comprehensively investigated the prognostic and immunological features of senescence related TME genes in HNSC. By leveraging these senescence related TME genes, we successfully developed a risk model to predict HNSC prognosis and immunotherapy response, which was robustly validated using external transcriptome datasets. These findings provided evidence for the role of senescence in the TME and highlighted the potential of senescence-related biomarkers as promising therapeutic targets.

## MATERIALS AND METHODS

### Data acquisition and processing

RNA-seq derived gene expression data (N=520) which transformed into transcripts per kilobase million (TPM), somatic mutation data (N=511), Human Methylation 450 data (N=528) and clinicopathologic data for HNSC were downloaded from the TCGA database using the R package ‘TCGAbiolinks’ (version 3.15) [34] Microarray gene expression data (GSE41613, N=167) from GEO database were obtained and processed to normalized matrix data by GEOquery R package and used as a validation dataset [35].

A total of 1,889 Tumor associated senescence (TAS) were selected based on well-established databases and published literature, including MSigDB (http://www.gsea-msigdb.org/gsea/msigdb/index.jsp), SenMayo gene set and, The Human Ageing Genomic Resources (HAGR) [12, 36]. The immune-related genes (N= 4,723) were downloaded from ImmPort database [37]. TME-related genes were obtained from several previously published studies and selected by removing duplicates [38]. The gene lists were expanded by searching for senescence related TME genes through network analysis with a gene-gene network since there may be genes that have not yet been investigated among the collected gene lists in this study. A gene-gene network is a weighted graph representing genes as nodes and connections between genes as edges. The edges were measured by the magnitude of the correlation coefficients, indicating the similarity between two genes in the network. Network-based label propagations were performed by employing graph-based semi-supervised learning (SSL) to investigate the potentially associated genes related to prognostic senescence, propagating label information on query nodes to unlabeled nodes along with edges [39].

### KHUMC cohort

Between January 2011 and January 2019, we enrolled 72 patients diagnosed with HNSC at the Kyung Hee University Medical Center (KHUMC) who received curative treatment. The cohort, referred to as the KHUMC cohort, was prospectively followed up for over 5 years following treatment, during which their clinical data, including age, sex, smoking history, and treatment type, were obtained. Furthermore, survival and recurrence information were retrospectively assessed for each patient.

Tissue samples were collected immediately after surgery or biopsy from patients diagnosed with HNSC. Total RNA was extracted from these samples using The TRIzol^®^ Reagent. To generate cDNA, the extracted total RNA was reverse transcribed using the Tetro cDNA Synthesis Kit (Bioline, London, UK) according to the manufacturer’s protocol. Subsequently, the isolated total RNA samples were sent to Applied Biosystems Macrogen Korea for sequencing, pre-processing, and transcriptome analysis. The concentration of total RNA was determined using Quant-IT RiboGreen^®^ (Invitrogen, #R11490). Only high-quality RNA preparations, with an RNA Integrity Number (RIN) greater than 7.0, were selected for RNA library construction. Each sample was independently used to prepare a library with 1 μg of total RNA, employing the Illumina TruSeq^®^ Stranded mRNA Sample Prep Kit (Illumina Inc., #RS-122-2101). The libraries were quantified using KAPA Library Quantification Kits for Illumina Sequencing platforms, following the qPCR Quantification Protocol Guide (Kapa Biosystems, Wilmington, MA, USA, #KK4854), and their quality was assessed using the TapeStation D1000 ScreenTape (Agilent Technologies, Palo Alto, CA, USA, #5067-5582). Indexed libraries were then submitted to an Illumina NovaSeq (Illumina Inc.), and Macrogen Inc. (Seoul, Korea) performed paired-end (2 × 100 bp) sequencing.

### Clustering analysis for senescence related TME subtypes and characterization

To identify genes with differential expression between tumor and normal samples, we employed the Limma R package. We further selected differentially expressed genes that showed prognostic significance using the univariate Cox regression analysis.

To cluster HNSC samples, we utilized ConsensusClusterPlus to construct a consistency matrix [40]. The expression data of genes linked to senescence related TME served as the basis for determining the molecular subtypes of the samples. The “hc” algorithm (hierarchical clustering) and “1-Pearson correlation” were chosen as the metric distance for conducting 500 bootstraps with each bootstrap iteration involved 80% of the patients in the training set. We performed a grid search ranging from 2 to 10 clusters to determine the optimal number of clusters. The optimal cluster was selected based on the cumulative distribution function (CDF) and consensus matrix.

To explore the relationship between senescence-related TME subtypes and senescence-associated biological functions across different samples, we conducted a single-sample gene set enrichment analysis (ssGESA) using the R package GSVA [41]. Subsequently, ssGSEA scores were calculated for each sample on various functions for comparison between the subtypes. To identify significant differences in gene mutations between the senescence related TME subtypes in the TCGA-HNSC dataset, we used the maftools R package (version 2.6.05). Based on the subtype, we categorized the original mutation annotation format (MAF) into two distinct groups. Tumor mutation burden (TMB) scores were computed for each patient within the two subtypes using somatic mutation data. For the analysis of methylation, TCGA-HNSC HumanMethylation450 data was used and preprocessed using the Limma R package (v3.46.0) with an FDR-corrected P-value of 0.01 and absolute log fold change > 0.5 to identify differentially methylated CpGs between subtypes. Genome annotation of these differentially methylated probes was based on the Illumina protocol. Metascape (https://metascape.org) analysis was performed to identify the pathways associated with a gene set.

To compare the difference in survival between the two subtypes, Kaplan-Meier survival analysis was performed using the ‘survival’ and ‘survminer’ R packages. The Kaplan-Meier curves were utilized to generate p-values and hazard ratios (HR) with 95% confidence intervals (CI) through log-rank tests. For visual representation of the p-values, HR, and 95% CI for each variable, a forest plot was constructed using the ‘forestplot’ R package.

### Senescence related TME risk model and validation

The TCGA-HNSC cohort was randomly partitioned, with 70% of the dataset assigned to the training set and the remaining 30% assigned to the testing dataset. The PSTGs were screened using LASSO regression (glmnet R package) [42]. Finally, the correlation coefficients for core genes were obtained to calculate the senescence related TME risk score.

The senescence related TME risk score is calculated by summing the product of the coefficient (X) and the corresponding gene expression level (X), where the set of genes *S* involved into risk score calculations was given:


$$\text{Risk}\;\text{Score}=\sum_{i\in S}X_{i}\times Y_{i},$$

The median senescence related TME risk score was used as the cutoff value. The test set, GEO cohort, KHUMC cohort, and the entire TCGA-HNSC set were stratified into high-risk and low-risk groups according to this cutoff value. Subsequently, the survminer R package was utilized to analyze overall survival. To assess the predictive ability of the senescence related TME risk score, receiver operating characteristic (ROC) curve analysis was conducted using the “survivalROC” R package.

### Estimation of the immune cell landscape, immunophenoscore and prediction of immunotherapy responsiveness

CIBERSORT utilized a gene expression signature matrix derived from purified immune cell populations to deconvolute the composition of complex tissue samples [43]. It leverages support vector regression and an artificial immune system-like algorithm to estimate the relative proportions of different immune cell types in HNSC sample. The accuracy of immune cell fractionation was considered significant when the CIBERSORT output achieved a p-value of less than 0.05. For each HNSC sample, the stromal and immune scores were estimated by applying the ESTIMATE algorithm to the normalized expression matrix [44]. Data on individual immunophenoscore (IPS) for HNSC patients were obtained by downloading from the Cancer Immunome Atlas (https://tcia.at/home).

To validate the predicted treatment responsiveness, we employed the TIDE algorithm, which utilizes gene expression profiles as a computational method for predicting the efficacy of immune checkpoint blockade [45]. We also calculated the M2 tumor-associated macrophages (TAMs), tumor-associated fibroblasts (CAFs), and myeloid-derived suppressor cells (MDSCs), the dysfunction score of tumor-infiltrating cytotoxic T lymphocytes (CTLs) (T cell dysfunction), and the exclusion score of CTLs by immunosuppressive factors (T cell exclusion) through TIDE analysis.

### Single-cell RNA-seq data

To understand the expression of STCGs in different cell types, we applied Tumor Immune Single Cell Hub (TISCH, http://tisch.comp-genomics.org/). TISCH is an online database dedicated to the TME and comprises a comprehensive collection of 76 tumor datasets encompassing 27 types of cancer, including single-cell transcriptome profiles comprising nearly 2 million cells. In our study, we focused on examining the expression patterns of the STCGs in HNSC sample. To accomplish this, we utilized GSE103322, a HNSC single-cell RNA sequencing dataset, which is part of the extensive data available in the TISCH database [46]. GSE103322 contained data of 5,902 cells derived from 18 patients with oral cavity tumor. We explored the expression of the STCGs in HNSC at single-cell level and identified the distribution of expression of senescence-related TME core genes in GSE103322. The expression of STCGs was collapsed by mean value, and the gene expression level displayed using UMAP and violin plots was quantified by the normalized values.

## Supplementary Material

Supplementary Table 1
